# Identification of Surrogates of Protection against Yersiniosis in Immersion Vaccinated Atlantic Salmon

**DOI:** 10.1371/journal.pone.0040841

**Published:** 2012-07-12

**Authors:** Andrew R. Bridle, Ben F. Koop, Barbara F. Nowak

**Affiliations:** 1 National Centre for Marine Conservation and Resource Sustainability, University of Tasmania, Tasmania, Australia; 2 Department of Biology, University of Victoria, Victoria, British Columbia, Canada; Indian Institute of Science, India

## Abstract

Simple cost-effective bacterins are the earliest and most successfully used commercial vaccines in fish. In particular, those prepared from *Yersinia ruckeri* have proven effective at controlling Enteric Red Mouth Disease (ERM) and yersiniosis in rainbow trout and Atlantic salmon, respectively. However, the emergence of outbreaks of ERM caused by atypical biotypes of *Y. ruckeri* and reports of vaccine failure resulting in mass mortality of hatchery Atlantic salmon has reinvigorated interest in vaccines against fish bacterial diseases. Therefore the objective of this study was to identify surrogates of protection against yersiniosis using cDNA microarray to characterise the response of host genes in the gills of unvaccinated and vaccinated Atlantic salmon challenged with *Y. ruckeri*. Differentially expressed genes were identified using two-way ANOVA and restricted to those with >2.5-fold change at *P*<0.05. Using cDNA microarray we identified the expression of 6 genes in response to infection and 4 genes associated with the protective host response to yersiniosis. Analysis by real-time PCR confirmed that three immunologically relevant genes, namely a cathelicidin (47-fold) and a C-type lectin (19-fold) increased in response to yersiniosis. Including collagenase (17-fold increase), an important tissue remodelling and repair enzyme, these genes represent 3 of 6 non-protective and/or pathological responses to yersiniosis. Genes associated with the protective host response included an immunoglobulin gene and a selenoprotein that showed significant fold changes (15-fold increases each), highlighting the importance of antibody-mediated protection against yersiniosis. These findings provide much needed knowledge of the host-pathogen interaction in response to bacterial infection and immunisation in fish. Significantly, we identified a transcriptional biosignature consisting of predominantly immune-relevant genes (14 up and 3 down-regulated) in the gills of Atlantic salmon after immersion vaccination and before bacterial challenge. This biosignature may be used as a surrogate of protection and therefore as a predictor of vaccine success against yersiniosis.

## Introduction

Enteric Red Mouth Disease (ERM) and yersiniosis are closely related fish diseases traditionally named according to the species primarily affected. Both cause bacterial hemorrhagic septicaemia and are caused by the Gram negative bacteria *Yersinia ruckeri*. ERM disease was first reported in rainbow trout in the Hagerman Valley, USA in the 1950s and was eventually termed ERM in 1975 by the Fish Health Section of the American Fisheries Society [1], [2]. The disease has since been reported throughout all trout farming regions in the Northern hemisphere and significantly impacts the culture of this species [3]. Notably, *Y. ruckeri* is now a ubiquitous pathogen that has been isolated from both fresh water and diseased fish from a wide geographical area including all the salmonid producing areas of the world [4]. In fact, it was this increasing geographical distribution of *Y. ruckeri* that likely led to the emergence of a similarly well-described disease yersiniosis, once colloquially called ‘salmon blood spot disease’ and is similarly caused by *Y. ruckeri*. Like ERM affecting rainbow trout in the Northern hemisphere, yersiniosis is capable of causing mass mortality. In 2007 approximately half a million juvenile Atlantic salmon in an Australian hatchery died over the course of a few months from yersiniosis despite having been vaccinated against yersiniosis [5]. Furthermore, in recent years there have been reported cases of *Y. ruckeri* causing disease in Atlantic salmon cultured in the major salmon producing countries of the Northern hemisphere [6].

Vaccination as a means of controlling ERM and yersiniosis is one of the most significant and successful health practices within the aquaculture industry proving that the use of antibiotics to control bacterial diseases is likely unnecessary. The first commercial ERM vaccine was licensed in 1976 and was produced as a bacterin prepared from formalin-killed whole cells of *Y. ruckeri*. Intraperitoneal vaccination with this bacterin conferred almost complete protection from Enteric Red Mouth Disease (ERM) in rainbow trout and until recently, yersiniosis caused by *Y. ruckeri* infection in Atlantic salmon. Similarly, high levels of protection were found when fish were immersed in the bacterin for a short duration, and immersion remains as the primary route of vaccination against ERM or yersiniosis. As a result of the high protective efficacy conferred by this vaccine it provides a useful vaccine model for the investigation of fish immune responses to bacterial diseases.

However, the emergence of outbreaks of ERM caused by atypical biotypes of *Y. ruckeri* and reports of vaccine failure resulting in mass mortality of hatchery Atlantic salmon from yersiniosis has reinvigorated interest in vaccines against fish bacterial diseases. Fortunately, both circumstances have or are being addressed by substituting strains of *Y. ruckeri* used to prepare the bacterin or by using modified immersion delivery [5], [6]. As the production of global aquaculture continues to increase it is likely that bacterin-based vaccines against other fish bacterial diseases will encounter similar issues and require modification and subsequent efficacy testing. However, manufacturers of these modified vaccines face ever growing scrutiny regarding animal welfare issues common in disease challenges [7]. In the present study our objective was to identify potential surrogates of protection to yersiniosis using cDNA microarray to characterise the differential response of host genes in naive unvaccinated and vaccinated Atlantic salmon experimentally challenged with *Y. ruckeri*. It is envisaged that such transcriptional biosignatures when used as surrogates of protection will offer a viable alternative to disease challenges satisfying both vaccine regulatory agencies and animal welfare concerns.

## Results and Discussion

### Vaccine Protective Efficacy

**Figure 1:**
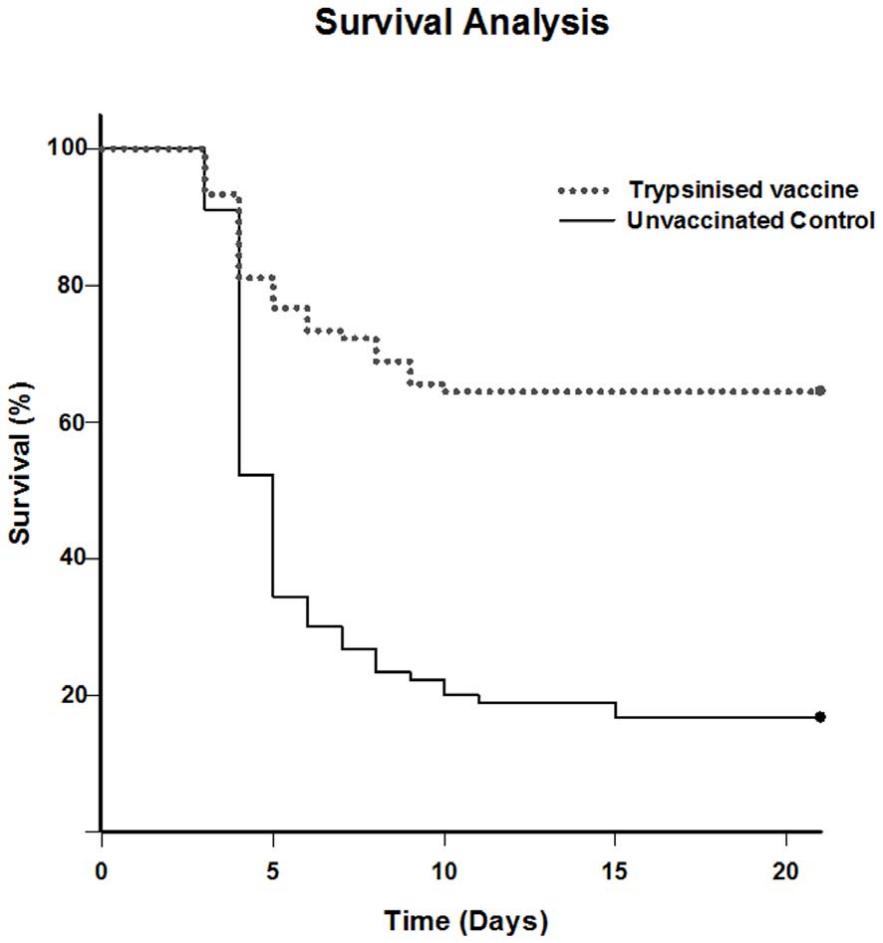
Survival analysis of naive unvaccinated and immersion vaccinated Atlantic salmon after experimental immersion challenge with *Y. ruckeri* at 6 weeks post-vaccination. Kaplan-Meier survival analysis of Atlantic salmon (n = 90) immersion vaccinated with trypsinised yersinivac-B showed that vaccinated salmon had statistically significantly (*P*<0.01) greater survival than naive unvaccinated salmon (n = 90) 21 day post-challenge. The vaccinated salmon had a relative percent survival (RPS) of 57% in relation to the 83% mortality of the control (unvaccinated) salmon.

Groups of 120 fish, immersion vaccinated with trypsinised yersinivac-B or unvaccinated controls were divided into 4 replicate tanks per treatment and challenged by immersion in fresh water containing *Y. ruckeri*. The yersiniosis challenge model has been previously shown to be a reliable and consistent disease model [8]. We chose to use a trypsinised preparation of yersinivac-B having previously reported on the improved efficacy of this vaccine [5]. In our study morbidity and or mortality as a result of infection was found to begin within 3–4 d post-challenge and had significantly subsided by 10–14 d post-challenge. Cumulative mortality 21 d post-challenge in the unvaccinated control group plateaued at 83% and was used to calculate the RPS survival of 57% in the vaccinated group. As expected Kaplan-Meier survival analysis clearly showed the survival of vaccinated fish to be statistically different to the unvaccinated fish (Fig. 1). Both gross and internal clinical pathology consistent with yersiniosis was identified in all fish that succumbed to the disease challenge and *Y. ruckeri* was isolated, cultured and identified by PCR from these fish. Likewise, PCR confirmed that *Y. ruckeri* was present in the kidneys of each fish sampled at 8 and 72 h post-challenge independent of vaccination status. This suggests that vaccine-induced protective responses do not prevent infection with *Y. ruckeri* but aid the clearance of the systemic infection as has been previously suggested in trout vaccinated against ERM [9] and Atlantic salmon vaccinated against furunculosis [10]. The impact this has on covert infection with *Y. ruckeri* (carrier status) in Atlantic salmon [11] and rainbow trout [12] remains unknown but may represent another potential measure of vaccine efficacy helping to reduce potential ERM and yersiniosis outbreaks in seemingly healthy fish.

### Differential Host Gene Expression Following *Y. ruckeri* Infection

Total RNA was extracted and reverse transcribed from the gills of uninfected unvaccinated (UU, see Table 1 for treatment definitions) Atlantic salmon and those that were unvaccinated and challenged with *Y. ruckeri* at 8 h (UI8h) and 72 h (UI72h) post-challenge. Microarray analysis using ANOVA compared the >2.5-fold differential gene expression of host genes between infected and uninfected salmon and identified 7 genes that were up-regulated 72 h post-challenge (Table.2). The differential regulation of genes at 72 h post-challenge in unvaccinated fish compared to uninfected unvaccinated fish was considered a non-protective/pathological response to *Y. ruckeri* infection as 83% of the group of fish in which these genes were identified died by 21 d post-challenge. The most significant of these genes were associated with innate immune responses including a cathelicidin gene identified by 2 different cDNA microarray probes that showed a 34.4 and 18.0-fold increase in expression at 72 h post-challenge, respectively. Cathelicidins are antimicrobial peptides (AMP) that exhibit strong antimicrobial activity against a broad range of pathogens in mammals, birds and fish in a dose dependent manner [13]. Real-time PCR was used to validate the microarray result and showed a 47.1-fold increase in expression of asCATH2 (genbank accession no. AY360357). The increased expression of this cathelicidin in the present study instigated our recently published investigation into the role of Atlantic salmon cathelicidins during yersiniosis and highlighted their potential multifaceted roles during infection [8]. Furthermore, this previous study similarly included real-time PCR of host expression during yersiniosis that further validates the results of our present microarray and real-time PCR analysis [8]. Previous studies in trout identified the differential expression of acute phase proteins (APPs) and pro-inflammatory genes following challenge with *Y. ruckeri* [14]–[18]. A recent study of Y. *ruckeri*-infected trout identified differential gene expression in the intestine and similarly found increased expression of APR and inflammatory-related genes including 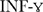 [19]. The suggestion by the authors that increased expression of 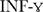 may be recruiting macrophages and initiating a cell-mediated response (CMR) has greater impact considering the recent finding by Ryckaert et al. [20] that *Y. ruckeri* is a facultative intracellular pathogen.

**Table 1:** Vaccination and challenge design.

	Pre-challenge (Uninfected)	Post-challenge 8 h (Infected)	72 h (Infected)
Naive **U**nvaccinated	6 fish (**UU**)	6 fish (**UI8h**)	6 fish (**UI72h**)
Immersion **V**accinated	6 fish (**VU**)	6 fish (**VI8h**)	6 fish (**VI72h**)

**Table 2:** Differentially regulated genes indicative of the non-protective/pathological response to *Y. ruckeri* infection.

cDNA microarrayprobe Acc. No.	cDNA microarrayUI72h vs UU (FC)	Real-time PCR UI72h vsUU (FC)	BLAST identification
EG852527	34.4 ↑	47.1 ↑	Cathelicidin (asCATH2)
CB511230	18.0 ↑	47.1 ↑	Cathelicidin antimicrobial peptide (asCATH2)
CB511048	6.4 ↑	19.5 ↑	C-type lectin domain family 4 member E
CK990871	6.3 ↑	16.7 ↑	Collagenase 3 precursor
CA046376	5.2 ↑	48.8 ↑	AF281355_1 differentially regulated trout protein 1
CA038364	3.9 ↑	NA	Cytochrome b-c1 complex subunit 9
EG845905	2.9 ↑	NA	45217 pfam05316, Mitochondrial ribosomal protein

The differential regulation of genes at 72 h post-challenge in unvaccinated fish (UI72h) compared to uninfected unvaccinated fish (UU) was considered a non-protective/pathological response to *Y. ruckeri* infection as 83% of the group of fish in which these genes were identified died by 21 d post-challenge. Arrows indicate the direction of the fold change while NA indicates genes not assessed by real-time PCR.

In the present study we identified a C-type lectin that increased 6.4-fold (microarray) and 19.5-fold (real-time PCR) to that of uninfected unvaccinated fish. Lectins are glycan-binding receptors that recognise glycan epitopes on pathogens and may opsonise them or activate complement leading to pathogen destruction. Like cathelicidins they are effectors in innate immunity and may have key roles in other aspects of immunity including leukocyte trafficking, cellular interactions and other immunological processes [21]. The immunohistochemical identification of a mannose binding lectin and the importance of innate immunity to protective responses in rainbow trout fry infected with *Y. ruckeri* has been suggested [14]. Likewise, bacterial infection caused by *Aeromonas salmonicida* induced the upregulation of multiple C-type lectins in the liver of Atlantic salmon [21]. More recently, nattectin, a C-type lectin identified in the fish *Thalassophryne nattereri*, when administered to mice was found to increase the production of pro-inflammatory cytokines that subsequently drive Th1 responses *in vivo* [22]. In the present study a potential lectin-induced Th1 response may contribute to host protection especially given the facultative intracellular nature of *Y. ruckeri* [20]. We also showed that collagenase 3 precursor, otherwise known as matrix metalloproteinase-13, was upregulated 6.3-fold (microarray) and 16.7-fold (real-time PCR) during yersiniosis. Belonging to the matrix metalloproteinase (MMP) family, collagenase-3 is involved in the breakdown of the extracellular matrix. A similar transcriptional profiling of channel catfish infected with *Flavobacterium columnare* identified a 67-fold increase of a MMP that shared 58% identity with collagenase 3 of *S. salar* [23]. In Japanese flounder kidney cells, lipopolysaccharide (LPS), that is often used as a substitute for Gram negative bacteria, induced the expression of inflammation-related genes that included collagenase [24]. This strongly suggests a role of tissue re-modelling in the gills of bacterially infected fish. Our results support those of Raida et al. [9] who found a strong correlation between ERM-induced genes relevant to innate immunity and *Y. ruckeri* load therefore suggesting that such responses were non-protective.

### Vaccine-induced Protective Host Gene Expression

Biosignatures indicative of a protective host response in immersion vaccinated fish following *Y. ruckeri* infection were identified from the differential host gene expression between unvaccinated fish 72 h post-challenge (UI72h) compared to vaccinated fish at the same time point (VI72h). Four differentially regulated genes were found to be associated with protection following vaccination and after challenge (Table.3). Two of these genes were undoubtedly related to immune functioning, the most significant of which was an immunoglobulin heavy chain (IgH) gene that showed a 15.2-fold (microarray) increased expression at 72 h post-challenge. The other was a selenoprotein that increased 4.2-fold (microarray) and 15.3-fold (real-time PCR) in vaccinated and infected fish. While relatively little is known of the role of selenoproteins in immunity it has been shown in mammals that they affect inflammation by regulating the oxidative state of immune cells [25]. Furthermore, knockout mice lacking T-cell selenoproteins have reduced antigen-specific production of immunoglobulins *in vivo* [26]. Our results suggest that Atlantic salmon selenoproteins play an important part in immune functions and possibly impact the specific antibody production to pathogens.

**Table 3:** Genes involved in vaccine-induced host protection.

cDNA microarray Probe Acc. No.	cDNA microarray VI72h vs UI72h (FC)	Real-time PCR VI72h vs UI72h (FC)	BLAST identification
EG849892	15.2 ↑	NA	*Salmo salar* IgH locus A genomic sequence*
DY701034	4.2 ↑	15.3 ↑	*Salmo salar* selenoprotein L (sell), mRNA*
DW560138	2.6 ↑	NA	60S ribosomal protein L37 [*Salmo salar*]*
EG851114	3.0 ↓	NA	UNKNOWN

* Previously annotated as unknown according to the cGRASP 32 K (Salmonid) cDNA annotation file (Nov. 2008). Blast identification shows the top BLASTX or BLASTN hit with highest total score and lower e-value.

Genes indicative of a protective host response in immersion vaccinated fish following *Y. ruckeri* infection were identified from the >2.5-fold differential host gene expression (ANOVA, P<0.05) between unvaccinated fish 72 h post-challenge (UI72h) compared to vaccinated fish at the same time point (VI72h). Arrows indicate the direction of the fold change while NA indicates genes not assessed by real-time PCR.

Specific antibodies are generally accepted to be important in vaccination, however, specific antibody-mediated protection against ERM or yersiniosis has never been clearly established. Numerous studies including ours have implicated that specific antibodies are important in vaccine-induced protection to ERM [9], [14], [19], [27]. Intriguingly, despite several attempts all but one, performed by Olesen [27], failed to passively immunise trout against ERM. Therefore confusion still exists concerning the role of specific antibodies in protection against this disease. A more recent study in unvaccinated trout using real-time PCR showed that expression of several inflammatory genes along with an IgM and IgT gene increased in the intestine following challenge with *Y. ruckeri* and were correlated with reduced bacteraemia [19]. Furthermore, immunohistochemistry showing a dense covering of IgT on the gills of rainbow trout fry infected with *Y. ruckeri* has been suggested as indicative of potential antibody-mediated protection during early life stages albeit prior to vaccination [14]. Interestingly, a recent study in trout found that plasma specific IgM antibody titers and bactericidal activity increased significantly following immersion vaccination against ERM and that significantly less *Y. ruckeri* were found in the blood of vaccinated trout 3–14 days post-challenge [9]. Targeted gene expression profiling of trout cytokine expression during ERM also suggested the importance of Th-1 like responses in vaccine-induced protection [28]. The potential contribution of other humoral or cellular factors to protection may explain repeated failures to demonstrate protection against ERM via passive immunisation. Moreover, it is likely that the recently identified ability of *Y. ruckeri* to reside within macrophages [20] may at least partially protect *Y. ruckeri* from antibody-mediated host protective responses. However, in the present study we identified no other immune-relevant genes in the vaccine-induced host protection and while not definitive our results support the view that vaccine-induced protection against ERM and yersiniosis is predominantly antibody-mediated. Furthermore, our results were obtained from the gills of Atlantic salmon, and as yet the contribution of organ-specific antibodies including potential mucosal antibodies towards protection against ERM or yersiniosis is similarly unclear.

### Surrogates of Protection Following Vaccination

**Figure 2:**
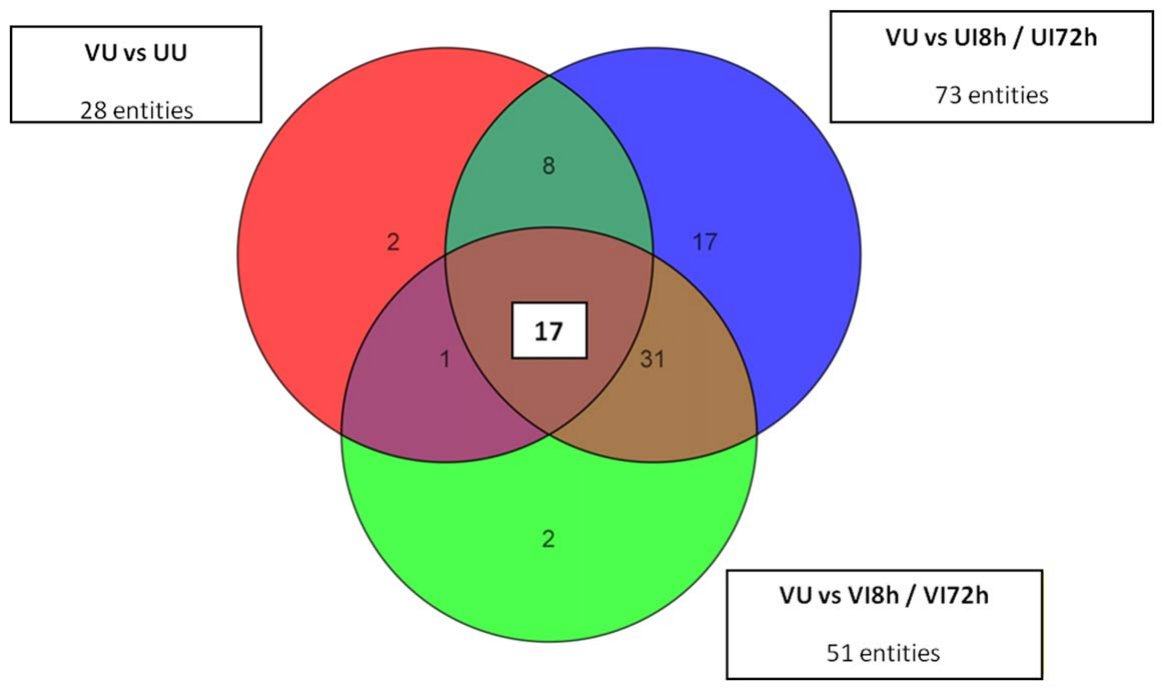
Venn diagram displaying differentially regulated genes in Atlantic salmon gill tissue that define vaccine success after vaccination but before challenge with *Y. ruckeri*. The highlighted central square shows the number of differentially expressed genes (ANOVA *P*<0.05 and >2.5-fold change) found in common between the comparison of the vaccinated unchallenged group of fish (VU) and all the other treatment/vaccination combinations at both time points post-challenge (UI8h/UI72h and VI8h/VI72h) and was considered as a specific vaccine-induced biosignature.

The primary aim of the study was to identify a biosignature that could act as a surrogate of protection and allow the efficacy of immersion vaccination to be predicted before challenge and therefore obviate the need for a disease challenge. Table 4 lists the differentially expressed genes found in common between the comparison of the vaccinated unchallenged group of fish (VU) and all the other treatment/vaccination combinations at both time points post-challenge (UI8h/UI72h and VI8h/VI72h) (Fig. 2.). Together these genes were considered a specific vaccine-induced biosignature. Furthermore, this biosignature was considered a potential predictor of vaccine-induced protection after vaccination but before *Y. ruckeri* challenge and therefore a surrogate of protection as reviewed and defined by Plotkin et al. [29]. Surrogates of protection following vaccination against ERM or yersiniosis have never been identified in fish. In the case or ERM or yersiniosis this is not unexpected considering the uncertainty surrounding the involvement of specific antibodies in vaccine-induced protection. Furthermore, although several transcriptional profiling studies have been performed in vaccinated and unvaccinated fish following infection [24], [30]–[36], other than an investigation into correlates of protection after vaccination against furunculosis [10] ours is the only other study to systematically identify genes of fish specifically related to protection following vaccination but before challenge and the first in ERM or yersiniosis.

**Table 4:** List of differentially expressed genes in the gills of Atlantic salmon identified as a potential surrogate of protection against yersiniosis.

cDNA microarrayProbeAcc. No.	VU vs UU(FC)	Real-time PCRVU vs UU(FC)	VU vs UI72h(FC)	VU vs VI72h(FC)	VU vsUI8h(FC)	VU vsVI8h(FC)	BLAST identification
EG929305	**3.4 ↑**	1.6 ↑	**2.5 ↑**	**2.8 ↑**	2.0 ↑	**3.2 ↑**	Uncharacterized protein KIAA1033
DY699380	**3.1 ↑**	2.4 ↑	2.1 ↑	1.7 ↑	1.8 ↑	**3.0 ↑**	LIM and actin-binding protein 1 [*Salmo salar*]*
DY729690	**2.7 ↑**	3.8 ↑	2.2 ↑	**2.6 ↑**	2.0 ↑	**3.2 ↑**	Hepcidin*
EG859007	**2.5 ↑**	1.5 ↑	**2.8 ↑**	2.2 ↑	2.3 ↑	2.2 ↑	E3 ubiquitin-protein ligase Itchy
CB514941	2.4 ↑	NA	**2.6 ↑**	1.9 ↑	**2.9 ↑**	**2.9 ↑**	UDP-N-acetylglucosamine transferase subunit
EG811438	2.3 ↑	NA	1.8 ↑	1.5 ↑	**2.8 ↑**	2.2 ↑	Olfactory receptor family C subfamily 4 member 11
DW553700	2.1 ↑	NA	**2.7 ↑**	1.8 ↑	2.1 ↑	2.3 ↑	MAK: Serine/threonine-protein kinase MAK
DY723439	2.0 ↑	NA	2.1 ↑	1.5 ↑	**2.6 ↑**	1.7 ↑	Sugar phosphate exchanger 2 [*Salmo salar*]*
DY738009	1.9 ↑	NA	**2.9 ↑**	1.9 ↑	**2.6 ↑**	1.9 ↑	Alcohol dehydrogenase class-3
CA054083	1.8 ↑	NA	**2.6 ↑**	2.3 ↑	**3.1 ↑**	**2.9 ↑**	Immunoglobulin mu heavy chain [*O. mykiss*]*
DY711765	1.8 ↑	NA	**2.7 ↑**	1.9 ↑	2.3 ↑	2.0 ↑	Lamina-associated polypeptide 2 isoforms
DW570661	1.7 ↑	NA	2.0 ↑	1.6 ↑	**2.5 ↑**	2.0 ↑	SAM and SH3 domain-containing protein 1
DY712739	1.7 ↑	NA	2.4 ↑	1.7 ↑	**2.6 ↑**	2.2 ↑	AY872256S1 *Oncorhynchus mykiss* IgH.A
DW565729	1.6 ↑	NA	**2.6 ↑**	1.9 ↑	2.0 ↑	2.3 ↑	Fish virus induced TRIM protein [*O. mykiss*]*
CB496376	1.7 ↓	NA	2.3 ↓	1.5 ↓	**2.9 ↓**	**2.9 ↓**	Myelin and lymphocyte protein
EG912256	2.0 ↓	NA	1.8 ↓	1.3 ↓	**2.6 ↓**	1.6 ↓	Bifunctional 3-phosphoadenosine 5-phosphosulfatesynthetase 2*
EG779342	1.8 ↓	NA	1.9 ↓	1.4 ↓	**2.6 ↓**	1.9 ↓	Thioredoxin interacting protein [*Salmo salar*]

* Previously annotated as unknown according to the cGRASP 32 K (Salmonid) cDNA annotation file (Nov. 2008). Blast identification shows the top BLASTX or BLASTN hit with highest total score and lower e-value.

The list of 17 differentially expressed genes (ANOVA P<0.05 and >2.5-fold change) found in common between the comparison of the vaccinated unchallenged group of fish (VU) and all the other treatment/vaccination combinations at both time points post-challenge (UI8h/UI72h and VI8h/VI72h) was considered as a specific vaccine-induced biosignature. Furthermore, this biosignature was considered a potential predictor of vaccine-induced protection after vaccination but before *Y. ruckeri* challenge and therefore a surrogate of protection. Arrows indicate the direction of the fold change, bold arrows indicate >2.5-fold change, while NA indicates genes not assessed by real-time PCR.

Potential correlates of protection in Atlantic salmon vaccinated against furunculosis included genes suggested to minimise the negative impacts of immune responses and to repair damages while no genes involved in the adaptive immune response were identified [10]. In contrast, along with genes involved in detoxification and repair namely alcohol dehydrogenase class-3 and thioredoxin interacting protein we identified the up-regulation of two Ig heavy chain transcripts within our protective biosignature. Other genes related to immune functioning included the antimicrobial peptide hepcidin also known to regulate iron homeostasis and reduce splenomegaly in mice [37] suggesting a potential role protecting against the haemolysis incurred during yersiniosis. The expression of lamina-associated polypeptide 2 isoforms also known as thymopoetin, E3 ubiquitin-protein ligase, SAM and SH3 domain containing protein 1, and myelin and lymphocyte protein were also regulated and have been implicated in mammalian T-cell development [38]–[40]. Furthermore, the regulation of thymopoetin, normally produced by the thymus, may indicate the involvement of the recently described thymus-like gill associated lymphatic organ [41]. Also related to immunity are a LIM and actin-binding protein known to regulate the internalisation of bacteria in mammals [42] and the fish virus induced TRIM protein also associated with immune recognition of pathogens and diverse receptors of the host immune system [43]. Our findings are consistent with transcriptional studies in other animal disease models that have identified biosignatures rather than a single marker as predictors of vaccine success [44]–[47]. Moreover, our results provide important insights into the vaccine-induced protective responses of fish to bacterial infection and we are currently assessing the application of this biosignature in predicting vaccine efficacy against yersiniosis in Atlantic salmon.

In summary this study used a cDNA microarray and real-time PCR to elucidate the transcriptional responses in the gills of naive Atlantic salmon and those immersion-vaccinated with a trypsinised bacterin vaccine against yersiniosis both before and after disease challenge. The aims were two-fold, namely to investigate the host-pathogen interaction during yersiniosis and to define biosignatures that could predict vaccine success before challenge and therefore act as a surrogate of protection. Significantly, we found differentially regulated genes in vaccinated fish that strongly suggests the importance of antibody-mediated protection following vaccination. Furthermore we identified a potential biosignature as a surrogate of protection that can now be confirmed in more targeted yersiniosis vaccine related studies. The potential to predict vaccine success is a highly attractive prospect especially considering the increasing pressure to use alternatives to disease challenges due to increasing animal welfare concerns.

## Materials and Methods

### Ethics Statement

All animal work was performed in strict accordance with the Australian Code of Practice for the Care and Use of Animals for Scientific Purposes and was approved by the University of Tasmania Animal Ethics Committee (AEC permit number: A0010335).

### Fish

Atlantic salmon (*Salmo salar*) weighing 2 g were obtained from a local hatchery (SALTAS, Wayatinah, Tasmania, Australia) and maintained in a semi-recirculating freshwater system at 15°C at the National Centre for Marine Conservation and Resource Sustainability (NCMCRS) Aquaculture Facility, University of Tasmania, Australia. The specific pathogen-free status of the fish was both assessed and confirmed from a subsample of fish by culturing kidney samples on blood agar plates and standard PCR [11] using DNA isolated from spleen and whole blood in an attempt to isolate and identify *Y. ruckeri*.

### Vaccine and Pathogen

A trypsinised version of yersinivac-B (MSD Animal Health, Australia), a commercially available vaccine against yersiniosis, was prepared from 0.5% formalin-killed whole cells at 1 × 10^10^ cells mL^−1^ following the method of Egidius and Andersen [48]. The trypsinised vaccine was prepared by Dr Jeremy Carson of the Fish Health Unit, Department of Primary Industries, Parks, Water and Environment (DPIPWE), Tasmania who similarly developed the unmodified yersinivac-B vaccine now produced by MSD Animal Health Australia.

A pathogenic strain (TCFB 2282) of *Y. ruckeri* O1b was supplied by Dr Jeremy Carson (DPIPWE) having been originally isolated from a mass mortality event from a Tasmanian hatchery in 2007. To ensure pathogenicity and obtain appropriate challenge doses the isolate was passaged via three consecutive pilot challenges (data not shown).

### Vaccination and Challenge

Fish were allowed to acclimate to the tanks for approximately 4 weeks until they achieved a 5 g average fish weight. Fish were both vaccinated and challenged via immersion to mimic the natural route of infection and maximise any possible benefit from the mucosal immune response. Immersion vaccination consisted of reducing the water level of the holding tank to 500 L and adding 1 L of one of a trypsinised preparation of yersinivac-B. The water volume of the holding tank containing control fish was similarly reduced; however, no vaccine or alternative chemical was added. The fish were maintained in 15°C fresh water for 6 weeks before immersion challenge with *Y. ruckeri*.

At six weeks post-vaccination fish (n = 120) from each treatment were randomly transferred to an infection room where they were challenged with *Y. ruckeri* O1b, strain TCFB 2282 at 4.3×10^6^ colony forming units (CFU) mL^−1^ for 1 h in air-saturated 20 L buckets containing 15°C fresh water. Having initially estimated the challenge dose using turbidity measurements, viable counts were determined by the Miles and Misra method [49] using TSA plates incubated for 48 h at 25°C. The challenged fish were then transferred into 200 L semi-recirculating tanks each sharing a common water supply but individually isolated by UV-disinfection. There were 4 tanks per treatment, each containing 30 fish, and moribund fish and any mortalities were removed from the tanks daily and used to calculate relative percent survival (RPS) using the formula RPS  =  (1− (% mortality/% control mortality)) × 100 [50]. At 21 days the challenge was terminated and survival data were analysed using Kaplan-Meier survival analysis.

### Sampling and Verification of *Y. ruckeri* Infection

At 0 h (pre-challenge), 8 h, and 72 h post-challenge six fish from both vaccinated and unvaccinated fish were euthanized and tissue dissected from the gill and stored in an RNA preservation reagent (25 mM sodium citrate, 10 mM EDTA, 4 M ammonium sulphate, pH 5.2) at −20°C until use (Table 1). Sampled fish were only taken from the fourth replicate tank for each treatment so as to avoid affecting the survival analysis which was conducted on the three remaining replicate tanks per treatment.

To verify the cause of death or morbidity of challenged fish kidney swabs from every moribund/dead fish were cultured on blood agar plates and standard PCR performed using primers specific to the 16S rRNA gene of *Y. ruckeri* [11].

### RNA Preparation and Microarray Hybridisation

Total RNA was extracted from sampled gill tissue stored at −20°C in an RNA preservation reagent using a tissue pulveriser cooled in liquid nitrogen. RNA from pulverised tissue samples was further extracted and purified using TRI Reagent (Molecular Research Center, OH, USA) including DNAse treatment (Turbo DNase, Ambion, TX, USA) and a Qiagen RNeasy column-based cleanup according to manufacturer’s instructions (Qiagen, VIC, Australia). Total RNA concentrations were determined using an Invitrogen Qubit fluorometer and Quant-iT RNA assay kit (Invitrogen, VIC, Australia). RNA quality was assessed for purity using a spectrophotometer (A_260/230_ and A_260/280_) and the integrity of the RNA was estimated from gel electrophoresis on a 1% agarose gel.

A two-colour microarray experiment using a universal reference design was performed using 32 k cDNA microarrays [51]. Reference aRNA was synthesized using 15 µg total RNA pooled from four samples from the six treatments using an Amino Allyl MessageAmp II aRNA amplification Kit (Ambion) as per manufacturer’s instructions. Reference aRNA samples were stored at −80°C as single-use aliquots prior to labelling. Amino allyl-modified cDNA was synthesized using a Superscript III Indirect cDNA Labelling System (Invitrogen). In brief, 10 µg total RNA from the gill of each individual fish was reverse transcribed using an anchored oligo(dT)_20_ primer in cDNA synthesis reactions incorporating amino allyl-modified nucleotides. Modified cDNA and aRNA were labelled with Mono-Reactive Cy5 and Cy3 dye, respectively (GE Healthcare, NJ, USA). Samples were purified with S.N.A.P. columns (Invitrogen), and quantity and specific activity were determined through spectrophotometry (NanoDrop 1000, Thermo Scientific).

Array hybridization was performed using a Tecan Pro HS 4800 Hybridization Station (Tecan, Männedorf, Switzerland) using a Gene Expression Wash Buffer Kit (Agilent, CA, USA) supplemented with triton X-102 as per manufacturer’s instructions. A total of 250 and 400 ng of aRNA and cDNA, respectively were combined and 12 µL 10× blocking agent (Agilent) added to the sample. Before sample loading 60 µL of 2× GEx hybridization buffer (Agilent) was added to the 60 µL sample and incubated at 80°C for 10 min and held at 65°C until loading. Following a 60°C wash with pre-hybridisation buffer (Agilent) 120 µL of the pre-heated sample mixture was injected onto the microarray. Hybridization occurred over 12 h at 60°C using the Tecan Pro HS 4800 set at low agitation frequency. Each sample was hybridised to one single microarray, and 6 biological replicates were used per treatment group (a total of 36 arrays). Following the incubation, arrays were initially washed at 26°C (GE wash buffer 1) then at 37°C (GE was buffer 2) as per Agilent protocol and the slides were dried with 37 psi nitrogen gas and kept dark in a low-ozone environment (≤5 ppb).

### Microarray Analysis

Array scanning was performed on a ScanArray Express (PerkinElmer, MA, USA; 5 µm resolution), adjusting the PMT gain for optimised visualisation of each image. Fluorescence intensity data and quality measures were extracted with ImaGene 8.0 (BioDiscovery, CA, USA). Array element identification and annotation was assigned by the cGRASP consortium (http://web.uvic.ca/cbr/grasp) [52]. The annotated gene list can be found at http://web.uvic.ca/grasp/microarray/array.html. Data normalization and analysis was performed using GeneSpring GX11 (Agilent). The array raw signal data were normalized using a per-slide, per-block intensity dependent Lowess normalization and a per-sample, per-gene baseline to median normalization. After normalisation and filtering for quality two-way analysis of variance (ANOVA) was applied to compare the mean expression levels between treatments. Data were considered significant at *P*<0.05 and were corrected for false discovery rates using the Benjamini Hochberg method. ANOVA combined with Tukey’s HSD multiple comparisons test was used to identify statistically differing groups at *P*<0.05. Further analyses were restricted to statistically significant genes with >2.5-fold change in expression between conditions. All data can be downloaded from the Gene Expression Omnibus at www.ncbi.nml.gov/geo/with the GEO accession number GSE36332.

### Quantitative Real-time PCR Analysis

Total RNA (2 µg) isolated from the from the gill of each individual fish as described above and stored at −80°C was reverse transcribed using Tetro Reverse Transcriptase (Bioline, NSW, Australia) with Oligo (dT)_18_ priming according to manufacturer’s instructions. Quantitative real-time PCR (qPCR) was performed on cDNA reverse-transcribed from total RNA as described above using SYBR Green chemistry using an iQ5 Real-time PCR Detection System (Bio-Rad, NSW, Australia). qPCR reactions consisted of 20 µl volumes using 2×SensiFast SYBR PCR master mix (Bioline) and forward and reverse primers (400 nM of each) and 2 µl of cDNA. Each gene was assayed in duplicate and a five step, four-fold dilution series of a pool of cDNA from all samples was included on the same plate to calculate amplification efficiencies. The amplification program was as follows: 95°C for 2 min to activate the DNA polymerase followed by 40 cycles of 95°C for 5 s, 55°C for 20 s and 72°C for 10 s. At the end of the cycling protocol melt curve analysis was run to ensure amplification specificity. mRNA expression levels were normalised using the mean expressions of three reference genes – elongation factor 1α (EF1a), β-actin, and RNA polymerase 2 (RPL2) which maintained stable expression, as determined by qBase Plus software (Biogazelle, Belgium). The qPCR data including statistical analysis (ANOVA) and fold change were analysed with qBase Plus software.
